# ALGORITHM FOR EARLY AMBULATION IN INDIVIDUALS UNDERGOING NON-CONVENTIONAL HIP ENDOPROSTHESIS

**DOI:** 10.1590/1413-785220253305e290840

**Published:** 2025-09-22

**Authors:** Emília Cardoso Martinez, Soraya Shouko Hiyama, Glauber Alvarenga, Eduardo Sadao Yonamine, Vera Lúcia dos Santos Alves

**Affiliations:** 1 Faculdade de Ciencias Medicas da Santa Casa de Sao Paulo (FCMSCSP), Sao Paulo, SP, Brazil.; 2 Faculdade de Ciencias Medicas da Santa Casa de Sao Paulo (FCMSCSP), Grupo de Oncologia Ortopédica, Sao Paulo, SP, Brazil.; 3 Faculdade de Ciencias Medicas da Santa Casa de Sao Paulo (FCMSCSP), Serviço de Fisioterapia, Sao Paulo, SP, Brazil.

**Keywords:** Arthroplasty, Arthroplasty, Replacement, Hip, Neoplasm Metastasis, Pathologic Fracture, Artroplastia, Artroplastia de Quadril, Metástase Neoplásica, Fraturas Espontâneas

## Abstract

**Introduction:**

The incidence of bone metastases has increased in recent years, driven by population aging and the efficacy of treatments for visceral-origin cancers. Bone metastasis ranks as the third most common type, with approximately one-third located in the proximal femur. Prosthetic hip replacement is a key treatment option, enabling immediate weight-bearing and resumption of patients’ normal activities.

**Objective:**

This study aims to develop an algorithm to encourage early ambulation in patients undergoing hip arthroplasty with non-conventional endoprostheses, targeting improved recovery and minimized complications.

**Method:**

The algorithm was designed by the multidisciplinary team of the Orthopedic Oncology Service at Irmandade da Santa Casa de São Paulo. It focuses on promoting early ambulation for hospital discharge, incorporating specific patient-guided milestones to ensure safe mobility progression while respecting clinical conditions.

**Results:**

The mean time to sitting position was 1.64 ± 0.66 days, while ambulation was initiated at 3 ± 0.42 days.

**Conclusion:**

The implementation of the algorithm demonstrated its efficacy in inpatient rehabilitation through personalized milestones. This approach promotes early ambulation and enhances patient confidence in mobility. ***Level of Evidence VI; Case series.***

## RESUMO

**Introdução:**

A incidência de metástases ósseas tem aumentado nos últimos anos, impulsionada pelo envelhecimento populacional e pela eficácia dos tratamentos para câncer de origem visceral. A metástase óssea é a terceira mais comum, com cerca de um terço localizada no fêmur proximal. A substituição protética do quadril é uma opção de tratamento importante, pois permite carga imediata e a retomada das atividades normais dos pacientes.

**Objetivo:**

O objetivo deste estudo é desenvolver um algoritmo para incentivar a deambulação precoce em pacientes submetidos à artroplastia de quadril com endoprótese não convencional, visando melhorar a recuperação e minimizar complicações.

**Método:**

O algoritmo foi elaborado pela equipe multidisciplinar do Serviço de Oncologia Ortopédica da Irmandade da Santa Casa de São Paulo. Ele foca na promoção da deambulação precoce para a alta hospitalar, incorporando metas específicas que orientam os pacientes, assegurando um progresso seguro na mobilidade e respeitando as condições clínicas.

**Resultados:**

O tempo médio de sedestação ocorreu em 1,64 ± 0,66 dias, enquanto a marcha foi iniciada em 3 ± 0,42 dias.

**Conclusão:**

A implementação do algoritmo demonstrou eficácia na reabilitação de pacientes, priorizando metas personalizadas. Essa abordagem promove a deambulação precoce e aumenta a confiança dos pacientes em sua mobilidade. ***Nível de Evidência VI; Série de casos.***

## INTRODUCTION

The incidence of bone metastases has increased in recent years with the aging of the population and the large number of people who are monitored after a cancer diagnosis. It is estimated that this number of cases will double in 50 years.^1–3^

Of the 44 million people worldwide, who are treated for cancer, 30 to 80% will develop bone metastases. Metastatic bone disease can cause pain, loss of function, and pathological fractures,^4,5^ with pain being associated with severity and being unresponsive to drug treatment, and with numerous questions regarding the pathophysiological mechanisms related to osteolysis and bone fragility.^6^

Pathological fractures usually occur in advanced stages of metastatic disease. Conservative treatment requires complete bed rest, and although surgical approaches carry risks, they may resolve cases and may be the sole treatment or an adjunct to radiotherapy and chemotherapy.^7–11^ Surgery not only relieves pain but also improves function and maximizes independence, which is beneficial even if the patient's overall prognosis is poor.^5^

Metastatic bone lesions are commonly located in the proximal femur, and with the modernization of adjuvant treatment, patients are surviving longer. Consequently, reconstructive options have also evolved to keep pace with this increase in survival.^3^ Preoperative assessment of patients with metastatic bone lesions in the hip region should be thorough and accompanied by a multidisciplinary team to guide treatment.^7^

The criteria described by Mirels^8^ are used to guide the treatment of bone metastases that present an imminent risk of fracture. The score is based on the nature of the injury, location, and size of the injury, pain assessment, and functional activity. The higher the score, the greater the risk of fracture. When the score is low, radiotherapy is chosen as the treatment, while lesions with a score of eight or higher are those that receive a recommendation for internal fixation.^1,7,8^

In patients undergoing surgical treatment, when there is a fracture of the proximal femur due to metastasis, studies report one-year survival rates ranging from 17% to 63%. There are reports of survival rates of up to five years in 23.1% of the total population. This longer survival rate has an impact on the choice of surgical material, which must be planned according to the patient's prognosis, maximizing costs and implant failures in order to choose materials with lower complication rates.^1^

Before any surgical procedure, the possibility of adjuvant treatment is studied to increase the success of the surgery. Radiotherapy has shown good results in reducing pain and preventing disease progression. There are even indications for low-dose radiotherapy, which does not prevent bone or soft tissue healing, provided that it is started between 10 and 14 days after surgery.^1,9^

Bisphosphonates are used to prevent the progression of metastasis in bone tissue and can be used in combination with other treatments, showing increased survival, a 15% reduction in the risk of fractures, and pain relief.^1,10,11^

The most commonly used methods for surgical correction of metastatic fractures are endoprosthetic reconstruction, intramedullary nailing, open reduction, and internal fixation. Surgical approaches are pretty effective in palliative strategies, but there's still no clear consensus on the best surgical treatment.^1,12^ When there's a lot of bone loss near the head or neck of the femur, endoprosthetic replacement is needed, and it's thought that endoprosthetic replacement gives better long-term results compared to intramedullary nails.^1,13,14^

One of the greatest benefits of prosthetic replacement is the possibility of immediate weight bearing and almost complete functionality of the limb. Dislocation is a worrying complication in this group of patients due to altered healing and loss of muscle mass typical of patients undergoing cancer treatment. Dislocation is less prevalent when the acetabulum is not affected, since in a hemiarthroplasty, the femoral head is larger; however, it is associated with residual pain and acetabular wear.^1,7^ Despite all this evidence on the early rehabilitation process of cancer patients undergoing endoprosthesis after hip fracture, we did not find any established protocols in the literature. Thus, we aim to develop an algorithm to encourage early and safe walking in individuals with bone metastasis undergoing unconventional hip arthroplasty.

## METHOD

### Algorithm Creation

The algorithm was developed in response to the need for early mobilization to ensure safe hospital discharge for patients with hip fractures resulting from bone metastases, with fixation established using hip endoprostheses.

The algorithm was divided into goals that could be daily or not, depending on the clinical condition of each individual. Progression occurs when there are no adverse events and the patient is stable enough to move on to the next goal. All events and patient progress should be documented in minute detail on the follow-up form.

The algorithm was developed based on the multidisciplinary team's previous experience, which involves monitoring patients in the postoperative period in a surgical ward that follows the hospital's rehabilitation process. All hospitalized patients are monitored based on goals established during multidisciplinary meetings, which observe the implementation of the protocol and discuss any ongoing difficulties. For each surgical case, the surgical team creates a timeline that starts on the first day after surgery (day zero) and continues until discharge from the hospital. All patients followed the same sequence of gait progression and received guidance on the procedures, restrictions, and goals to be achieved at each stage. After surgery, patients continued to be monitored using traditional care and adhering to the guidelines outlined in the algorithm.

After creating the algorithm presented in the results section, a study was conducted using a series of cases that included 25 patients being monitored at the Orthopedic Oncology outpatient clinic of the Irmandade da Santa Casa de Misericórdia de São Paulo (ISCMSP). All participants were included after signing an informed consent form, and the cohort was established from January 2021 to January 2023. The Research Ethics Committee approved the study under CAAE 40326220.8.0000.5479.

The patients included were those over 18 years of age, of both sexes, followed up for bone metastasis and who underwent unconventional hip arthroplasty after hip fracture.

Patients with an imminent risk of fracture with a Mirels score > 8,^8,15^ were excluded, as were patients who had undergone any other type of surgical approach for the treatment of pathological fractures due to bone metastases, those with complications requiring surgical reintervention within six months after the initial surgery, those with cognitive impairment, neuromuscular disease, and/or any need for urgent or elective surgical intervention during the protocol.

The descriptive data of the patients followed up were tabulated, and statistical analysis was used to relate the variables in terms of mean and standard deviation, with correlations of patterns identified using the SPSS program, with statistical significance set at ***p*** ≤ 0.05.

## RESULTS

**Figure 1:**
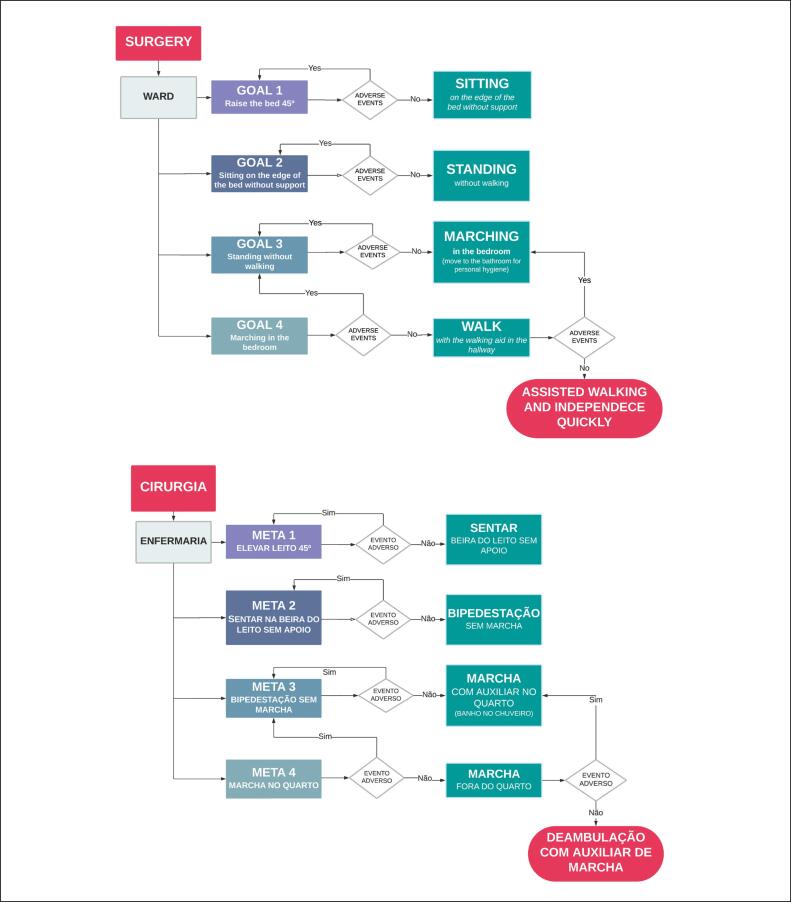
Algorithm for establishing safe walking in the immediate postoperative period of individuals with bone metastasis undergoing hip replacement.

The algorithm begins with the decision to proceed with hip replacement surgery and continues with patients being monitored in the orthopedic ward at the ISCMSP Central Hospital. (Figure 1) The algorithm proceeds by establishing sequentially numbered goals that must be followed in numerical order at each stage. All patients should be monitored by a multidisciplinary team, which should guide care at each stage of the algorithm and provide support for transfers. To further understand the algorithm, the goals will be explained separately as shown.

- Goal 1 – Objective: Raise the bed to 45°. In the immediate postoperative period, the patient is referred to the ward. Upon arrival, the bed should be raised to 45°. After observation, if no adverse events occur, the patient may progress to sitting on the edge of the bed without support. In the event of any adverse event, the patient should return to bed and maintain an elevation of 45°.
- Goal 2 – Objective: Sitting on the edge of the bed without support. With the bed at a 45° angle, the patient should be encouraged to sit on the edge of the bed without support. If there are no adverse events, the patient may continue with the goal of standing without walking. If the patient experiences any adverse events, they should return to a sitting position at the edge of the bed without support.
- Goal 3 – Objective: Standing without walking. Initially, the patient should be encouraged to stand without walking. A member of the multidisciplinary team should assist with all transfers. If there are no adverse events, the patient may begin walking with the therapist and using a walking aid in the room to move to the bathroom for personal hygiene. If the patient experiences any adverse events, they should return to standing without walking.
- Goal 4 – Objective: Marching in the bedroom. Initially, the patient should stand without walking and, with the aid of a walking aid, should be able to move around for personal hygiene. If there are no adverse events, the patient will be encouraged to walk with the walking aid in the hallway. If the patient experiences any adverse events, they should return to walking in the room.

The goals outline the sequence of actions to enable the patient to return to assisted walking and independence quickly. The 25 patients who followed the established algorithm have the profile shown in Table 1.

**Table 1:** Characterization of the sample, with 25 patients evaluated and classified according to their primary tumor site.

Variables	n=25 (total)	%
Women	15	60
Between 40 and 49 years old	03	12
Between 50 and 59 years old	09	36
Between 60 and 69 years old	07	28
Between 70 and 79 years old	03	12
Between 80 and 89 years old	03	12

The primary diagnosis of breast cancer was present in 32% of cases, followed by multiple myeloma (28%), prostate and kidney (12% each), thyroid with 8%, and lung or liver cancer in 4%, respectively.

Table 2 presents the average lengths of pre- and post-operative hospitalization, as well as the time in days required to achieve ambulation. There were no complications that required discontinuation of the protocol in the 25 cases analyzed. The goals were achieved slowly and cautiously, prioritizing patient safety and minimizing the risk of falls during hospitalization. This extremely cautious approach was crucial in ensuring that all patients remained stable and able to continue rehabilitation, as a fall in the ward could seriously compromise their future mobility.

**Table 2:** Average length of stay (pre- and post-operative) and time to achieve ambulation.

Variables	Average (SD)
Days of preoperative hospitalization	09(5.7)
Days of hospitalization POS OP	04(1.22)
Days for sitting	1.64(0.66)
Days for walking	03(0.42)

## DISCUSSION

The creation of an algorithm for early walking is not intended to categorize and generalize individuals, but rather to transform the acquisition of walking into a natural act without barriers. In this study, we aimed to create the algorithm and test its application based on the fracture risk classification according to Mirels^8^ criteria, observing that all individuals submitted to the algorithm had no lower limb load restrictions to start the rehabilitation process.

The concern for creating the algorithm arose from observing the short-term mortality rate of patients undergoing surgical treatment for proximal femur bone metastasis. Statistics show that within 30 days postoperatively, bed rest is associated with death from pneumonia and pulmonary embolism.^16^ To reduce mortality in these patients, it is recommended that surgery be performed within 48 hours of hospital admission and that early mobilization be initiated. The surgical approach to treating pathological fractures caused by bone metastasis allows for early mobilization, thereby preventing postoperative complications in these patients.

The length of hospitalization can be a significant factor in evaluating the overall recovery process and the utilization of healthcare resources for patients undergoing this type of surgery. A shorter hospital stay may indicate better postoperative recovery and fewer complications.^17^

Physical therapy should begin immediately after surgery to help with balance, walking, and muscle strength, allowing patients to return to their normal activities as soon as possible.^18^ Immobilization has been linked to higher death rates and worse outcomes for these patients, and it's also been found that waiting to start walking increases the risk of pneumonia.^18^

Postoperative physical therapy reduces the incidence of pressure ulcers and improves balance, muscle strength, gait, function, and independence when initiated early, within 48 hours after surgery. It also reduces the length of hospital stay for these patients, promoting ambulation outside the hospital environment, while also increasing strength and balance. These patients tend to be less afraid of falling after hospital discharge, thus enabling them to accelerate their functional recovery, resulting in a decrease in dependence after discharge.^18,19^ Based on this information, we identified the need to develop a method to minimize mortality and enhance the functionality of these patients after surgery. Initially, we set daily goals with the objective of hospital discharge within three or four days. However, due to clinical and orthopedic changes, we need to respect individual timing and encouragement, shifting the algorithm to goals rather than days, thereby optimizing the postoperative approach in an individualized manner for each patient's response. The goals, as can be seen in the algorithm, have a sequence, but there is no set time frame for their achievement. The last goal may be reached on the first day after surgery, provided that the patient achieves all previous goals without any adverse events.

After applying the algorithm, we observed a fairly consistent pattern for sitting up. All individuals were able to sit up before the third day of hospitalization. Still, not all were able to walk independently with assistance at the time of discharge, due to individual clinical and postoperative conditions.

An algorithm is a set of instructions or sequential steps designed to solve a problem or perform a specific task. It provides a series of clear and organized steps that help guide the walking process for hospitalized patients. It is a type of visual guidance that facilitates clinical decision-making based on different criteria and patient conditions. This study may contribute to reducing the length of hospital stay for patients with bone metastases in the proximal femur who undergo unconventional endoprosthesis surgery, thereby preventing complications such as pneumonia and decreasing muscle strength, among other benefits already mentioned in this study. With shorter hospital stays, this algorithm will enable a reduction in mortality rates and complications in the immediate postoperative period for these patients, also reducing functional loss, which is essential for patients to return to their normal activities.

## CONCLUSION

The implementation of the early gait algorithm proved to be an effective approach in patient rehabilitation. By prioritizing personalized goals and respecting each individual's pace and clinical condition, this strategy enables recovery tailored to each person's specific needs. Promoting early ambulation is essential, as it helps prevent complications such as pneumonia and pulmonary embolism, while also facilitating a quicker return to daily activities. This approach not only reduces hospitalization time but also strengthens patients’ confidence in their ability to move independently, promoting a safer and more efficient recovery. The results suggest that adopting personalized rehabilitation goals, rather than strict deadlines, makes the postoperative process more flexible and effective, bringing significant benefits to patients’ well-being in the medium and long term.
